# *N*-methyl-D-aspartate receptor activation mediates lung fibroblast proliferation and differentiation in hyperoxia-induced chronic lung disease in newborn rats

**DOI:** 10.1186/s12931-016-0453-1

**Published:** 2016-10-21

**Authors:** YanRui Wang, ShaoJie Yue, ZiQiang Luo, ChuanDing Cao, XiaoHe Yu, ZhengChang Liao, MingJie Wang

**Affiliations:** 1 Department of Neonatology, Xiangya Hospital, Central South University, Xiangya Road 87, Changsha, 410008 China; 2 Department of Physiology, Xiangya Medical College, Central South University, Changsha, 410008 China; 3 Department of Neonatology, Heze Municipal Hospital, Heze, 274000 China

**Keywords:** Hyperoxia, *N*-methyl-D-aspartate receptor, Newborn, Lung fibroblast, Chronic lung disease

## Abstract

**Background:**

Previous studies have suggested that endogenous glutamate and its *N*-methyl-D-aspartate receptors (NMDARs) play important roles in hyperoxia-induced acute lung injury in newborn rats. We hypothesized that NMDAR activation also participates in the development of chronic lung injury after withdrawal of hyperoxic conditions.

**Methods:**

In order to rule out the anti-inflammatory effects of NMDAR inhibitor on acute lung injury, the efficacy of MK-801 was evaluated in vivo using newborn Sprague-Dawley rats treated starting 4 days after cessation of hyperoxia exposure (on postnatal day 8). The role of NMDAR activation in hyperoxia-induced lung fibroblast proliferation and differentiation was examined in vitro using primary cells derived from the lungs of 8-day-old Sprague-Dawley rats exposed to hyperoxic conditions.

**Results:**

Hyperoxia for 3 days induced acute lung injury in newborn rats. The acute injury almost completely disappeared 4 days after cessation of hyperoxia exposure. However, pulmonary fibrosis, impaired alveolarization, and decreased pulmonary compliance were observed on postnatal days 15 and 22. MK-801 treatment during the recovery period was found to alleviate the chronic damage induced by hyperoxia. Four NMDAR 2 s were found to be upregulated in the lung fibroblasts of newborn rats exposed to hyperoxia. In addition, the proliferation and upregulation of alpha-smooth muscle actin and (pro) collagen I in lung fibroblasts were detected in hyperoxia-exposed rats. MK-801 inhibited these changes.

**Conclusions:**

NMDAR activation mediated lung fibroblast proliferation and differentiation and played a role in the development of hyperoxia-induced chronic lung damage in newborn rats.

## Background

The introduction of prenatal steroid use, surfactant treatment, new ventilation strategies, and other treatments has resulted in major improvements in the reduction of high-concentration oxygen inhalation in newborns over the last 40 years. However, oxygen is still the most commonly used therapy in neonatal nurseries as an integral part of respiratory support. Additionally, in preterm infants, transition at birth from a relatively hypoxic environment in utero to room air inevitably represents a hyperoxic event. Exposure of the developing lung to hyperoxia is a critical factor in the occurrence of bronchopulmonary dysplasia (BPD) [1, 2].

We previously demonstrated that large amounts of endogenous glutamate were released into the lungs of newborn rats exposed to hyperoxic conditions for 1 to 3 days; this phenomenon was found to be unrelated to nonspecific acute lung injury and airway inflammation [3]. Glutamate, a major excitatory neurotransmitter that is abundantly present in the mammalian central nervous system (CNS), plays key roles in brain development, learning and memory, and synaptic plasticity [4]. However, glutamate may also exert lethal effects on neurons through the overactivation of *N*-methyl-D-aspartate receptors (NMDARs) [5]. Overstimulation of NMDARs leads to neuronal cell death in several acute and chronic conditions [6]. Previous studies strongly suggest that endogenous glutamate and NMDARs play important roles during acute lung injury and airway inflammation [7–10]. Moreover, lung NMDARs are strongly expressed after a 3–7-day exposure to hyperoxia [3], and the NMDAR antagonist MK-801 was shown to decrease hyperoxia-induced acute lung damage [3, 11]. These results indicated that glutamate and its NMDAR play important roles in hyperoxia-induced acute lung injury.

Pulmonary inflammation induced by the high levels of oxygen free radicals during hyperoxia represents one of the most important mechanisms underlying hyperoxia-induced BPD [12]. Studies have shown that higher levels of collagen deposition and alveolarization damage in the lung are associated with more severe acute lung injury [13, 14]. However, severe acute lung injury and inflammatory reactions are not observed in most preterm patients suffering from “new” BPD [1, 2]. “New” BPD is considered a developmental disease resulting from an interference or interruption in the growth of the lung [2]. Studies have shown that exposure to 100 % hyperoxia for 1 to 3 days induces a model of “new” BPD, which manifests as moderate lung inflammation and delayed alveolarization during recovery in newborn rats [15, 16]. Acute inflammation is not the essential cause of chronic lung disease (CLD) induced by hyperoxia; however, the mechanism underlying the development of CLD following hyperoxia is still unclear.

Fibroblasts of the fetal and neonatal lung are important interstitial cells in normal and abnormal lung function and development [17]; therefore, these cells play important roles in the development of CLD in infants. The principal role of lung fibroblasts is to maintain the integrity of the alveolar structure via the synthesis, secretion, maintenance, degradation, and remodeling of the extracellular matrix (ECM) [18]. Tissue injury and repair follows a defined pathway: fibroblasts migrate into the provisional matrix, proliferate, and produce additional ECM components, such as fibronectin and collagen, resulting in the accumulation of fibroblasts within granulation tissue [19, 20].

In 1980, Gray et al. first found that 30 mM L-glutamate exerts toxic effects on fibroblasts in the context of Huntington’s disease [21]. Glutamate induces cellular degeneration in fibroblasts from patients with Huntington’s disease and in normal skin fibroblasts [22–24]. Early structure-activity studies have established that the structure of glutamate is ideal for the activation of NMDARs. In the last few years, NMDA-type glutamate receptors have been detected in fibroblasts, including human fibroblast-like synoviocytes [25], NIH3T3 mouse fibroblasts [26], and human periodontal ligament fibroblasts [27]. In our previous report demonstrating the abundant release of glutamate in hyperoxia-induced acute lung injury in newborn rats [3], our data suggested that lung fibroblasts may be excited by endogenous glutamate via NMDAR activation. However, to date, no studies have focused on the expression and functions of NMDARs in lung fibroblasts.

In this work, we hypothesized that the activation of NMDAR may regulate the proliferation and differentiation of lung fibroblasts and may participate in hyperoxia-induced CLD in newborn rats. Using a model of lung injury in 3-day-hyperoxia-induced newborn rats, the effects of NMDAR inhibitor treatment during the chronic lung injury phase, following the withdrawal of hyperoxic conditions, were evaluated in vivo. The role of NMDAR activation in hyperoxia-induced lung fibroblast proliferation and differentiation was examined in vitro using primary cell cultures derived from the lungs of newborn rats during the chronic injury phase.

## Methods

### Animals and study design

Timed pregnant Sprague-Dawley rats were purchased from the Animal Center of Central South University, Changsha, China. Pups were delivered naturally at full term (22 days). All pups were maintained in room air for the first 2 h of life before randomization into four study groups: a) air + normal saline (control group, C); b) air + MK-801 (M); c) hyperoxia + normal saline (H); and d) hyperoxia + MK-801 (H + M). Hyperoxia-exposed animals were placed in 95 % oxygen conditions for 72 h and allowed to recover in room air for the next 18 days. On postnatal days 8 to 10, the pups were intraperitoneally injected with 0.05 mg/kg MK-801 per day for a total of 3 days; corresponding control groups were treated with the same volume of normal saline on the same days. Animals in each group were killed on postnatal days 4, 8, 15, and 22 by intraperitoneal injection of pentobarbital (50 mg/kg body weight), and lung tissue was harvested for analysis. A total of 384 pups were used in vivo study, including six pups at each time point for each method in each group. Samples from each animal were used for only one type of analysis.

### Primary lung fibroblast culture and drug treatment

Six randomly chosen pups from the 3-day hyperoxia and room-air control group were sacrificed on postnatal day 8. The lungs were removed, minced into 1-mm^3^ pieces, and dissociated in Hanks buffered saline solution (HBSS; Hyclone, China) containing DNAse (20 μg/mL) and 0.25 % trypsin at 37 °C for 10 min. Dissociation was stopped by adding Dulbecco’s modified Eagle’s medium (DMEM; Hyclone) with 10 % charcoal-stripped fetal calf serum (FCS^−^). The cells were filtered through a sterile 45-μm cell strainer and centrifuged at 650 × *g* for 10 min at 4 °C. The cell pellets were resuspended in DMEM with 10 % FCS^−^, and cells were plated at 6 × 10^6^ cells/dish in 100-mm^2^ culture dishes for 60 min at 37 °C to allow lung fibroblast adherence. When fibroblasts reached 90 % confluence, the cells were serum-starved for 24 h in serum-free medium. mRNA transcripts corresponding to the N-methyl D-aspartate subunit 1 (*NR1*) gene and four NR2 subunits (*NR2*s) from the hyperoxia and control groups were measured directly. Cell proliferation and expression of type I procollagen (PC I) and alpha-smooth muscle actin (α-SMA) were measured after treatment with 0.5 mM MK-801 or the same volume of saline for 24 h.

### Study measurements

#### Collection and analysis of bronchoalveolar lavage fluid (BALF)

On postnatal days 4, 8, 15, and 22, pups from each group were sacrificed by administration of intraperitoneal pentobarbital (50 mg/kg). After tracheal intubation and injection of ice-cold 0.9 % saline (0.035 mL/g × g body weight, five times), BALF was pooled, and the total volume was recorded. More than 85 % of the instilled saline was collected from each animal. Total cells in BALF were counted using a CASY-1 cell counter (RJF Sales, Scotch Plains, NJ, USA). BALF samples were then spun at 400 × *g* for 5 min, and supernatants were aliquoted and frozen at -80 °C. Total protein (TP) content in BALF (as an index of protein leakage due to alveolar microvascular membrane injury) was measured using Lowry assays. Lactate dehydrogenase activation (LDH) was assayed as an index of respiratory membrane barrier damage and cellular membrane injury, using an LDH assay kit (Nanjing Jiancheng Bioengineering Institute, Nanjing, China) according to the manufacturer’s protocol.

### Lung wet weight and dry weight

Lung specimens were obtained from random samples of different groups on postnatal days 4, 8, 15, and 22. After the wet weights were measured, the tissues were placed in an oven and maintained at a temperature of 80 °C for 72 h. After the dry weight of the lung was measured, the presence of pulmonary edema was examined by determination of the lung wet-weight/dry-weight ratio (W/D).

### Lung hydroxyproline (HYP) assays

Since lung HYP is almost exclusively derived from collagen, whole-lung collagen content was estimated by measurement of HYP levels. The whole pulmonary lobes were dissected free from their bronchi and blood vessels. The wet weights of the whole right lungs were measured, and the lungs were then homogenized. HYP content in lung hydrolysates was determined using an HYP assay kit (Nanjing Jiancheng Bioengineering Institute).

### Measurement of dynamic lung compliance (Cdyn)

Low pulmonary compliance indicates a stiff lung and can be thought of as a thick balloon; this parameter has been widely used in the evaluation of lung development in neonatal patients with CLD [28–31]. To determine the functional impact of our histological findings, we further performed dynamic lung compliance testing. Measurements were performed using the Buxco system (Buxco Research Systems, Wilmington, NC, USA). At postnatal day 22, rats of each group were anesthetized with 10 % chloral hydrate (0.5 mL/kg) and tracheostomized. The Cdyn (mL/cmH_2_O), which reflects the change in pulmonary elastic resistance, was calculated according to the recorded variance in thoracic pressure in the esophagus and the respiratory flow.

### Lung histology

Rats were sacrificed on postnatal days 4, 8, 15, and 22. The lungs of a random sample from each group were inflated with 4 % paraformaldehyde in PBS via polyethylene catheters in the trachea, at a pressure of 23 cm H_2_O [32]. The lungs were then fixed in 4 % paraformaldehyde and embedded in paraffin. Five-micron-thick sections of the lung were stained with hematoxylin and eosin. Lung sections from all lungs were examined for histological changes. All histological evaluations were performed by an independent pathologist who was unaware of the experimental groups.

### Radial alveolar count (RAC)

One of the hallmarks of CLD in neonates is simplified distal airspaces, representing arrest of lung development. RAC, an objective measure of alveolar number, was used to assess lung development, as previously described [33, 34]. Respiratory bronchioles were identified as bronchioles lined by the epithelium in one part of the wall. From the center of the respiratory bronchiole, a perpendicular line was dropped to the edge of the acinus connective tissue, septum, or pleura, and the number of septa intersected by this line was counted. At least 15 counts were performed for each animal.

### Analysis of genes expression by reverse transcription quantitative polymerase chain reaction (RT-qPCR)

Total RNA were isolated from the samples of pulmonary tissues and primary lung fibroblast using Trizol Reagent (Sigma, St. Louis, MO, USA) according to the manufacturer’s instructions. Reverse transcription was performed using 1 μg total RNA and oligo (DT) primers in a 20-μL reaction, according to the manufacturer’s protocol (PE Applied Biosystems, Foster City, CA, USA). Relative quantitative SYBR Green-based real-time PCR was performed (Shinegene Molecular Biology Technology Ltd., Shanghai, China) with the actin gene as an endogenous control. The primers were designed using Primer Express version 1.0 software (Table 1). mRNA levels of type I procollagen for pulmonary tissues, and those of *NR1* and four *NR2*s, as well as type I procollagen and α-SMA for lung fibroblast, were measured.

**Table 1:** Polymerase chain reaction primer sequences

Primer name	Primer sequence
NR1	f.CAGGAGTGGAACGGAATCAT r:ACTTGAAGGGCTTGGAGAAC’
NR2A	f:AGCCATTGCTGTCTTCGTTT r:ATCTTGCTGGTTGTGCCTTT
NR2B	f:GCGATAATGGCGGATAAGGA r:AGGTAGGTGGTGACGATGGAA
NR2C	f:CACACCCACATGGTCAAGTTC r:ATGGTGACCAGCTTGCAGC
NR2D	f:CGAGGATGGCTTTCTGGTGA r:ATACTTGAGGCGGAGGGTCTG
PC I	f:CCATCAAGGTCTACTGCAACATG r:CATCGGTCATGCTCTCTCCAA
α-SMA	f: ACTGGGACGACATGGAAAAG, r:CATCTCCAGAGTCCAGCACA
β-Actin:	f:TGACGTGGACATCCGCAAAG r:CTGGAAGGTGGACAGCGAGG

NR1: NMDAR 1; NR2 A: NMDAR 2A; NR2 B: NMDAR 2B; NR2 C: NMDAR 2C; NR2 D: NMDAR 2D; PC I: type I procollagen

### Analysis of type I collagen by enzyme-linked immunosorbent assays (ELISAs)

The type I collagen levels in cell homogenates were measured using ELISA kits from Boster Biotechnology Co. (Wuhan, China), according to the manufacturer’s protocols.

### Analysis of α-SMA by western blotting

Total protein was extracted from cells using RIPA buffer, according to the manufacturer’s protocol (Santa Cruz Biotechnology, Santa Cruz, CA, USA). Protein concentrations were measured by BCA protein assays using a commercial kit from Pierce Biotechnology Inc. (Rockford, IL, USA). Total proteins (50 g/sample) were fractionated by sodium dodecyl sulfate polyacrylamide gel electrophoresis (SDS-PAGE) on 4–12 % Tris-glycine precast gradient gels (Invitrogen, Carlsbad, CA, USA), transferred to nitrocellulose membranes (Amersham, Piscataway, NJ, USA), and incubated with monoclonal anti-α-SMA and anti-β-actin antibodies (Cell Signaling Technology, Beverly, MA, USA) at 4 °C for 12 h. Thereafter, membranes were incubated with horseradish peroxidase (HRP)-conjugated anti-rabbit IgG (1:5000) for 60 min at 24 °C. The reactions were visualized using enhanced chemiluminescence and detected on a photographic film. The intensities of protein bands were quantified using a Quantity One Imaging Analysis Program (Bio-Rad, Hercules, CA, USA). The relative level of protein was measured by determining the ratio of α-SMA to β-actin.

### Cell proliferation assay

Cell proliferation was measured by 5-ethynyl-29-deoxyuridine (EdU) assays using an EdU assay kit (Ribobio, Guangzhou, China) according to the manufacturer’s instructions. Briefly, the cells were cultured in triplicate at 5 × 10^3^ cells/well in 96-well plates and treated as described. Cells were then exposed to 50 mM of EdU for additional 2 h at 37 °C. Next, cells were fixed with 4 % formaldehyde for 15 min at 24 °C and treated with 0.5 % Triton X-100 for 20 min at 24 °C for permeabilization. After three washes with phosphate-buffered saline (PBS), cells were treated with 100 μL of 1× Apollo^R^ reaction cocktail for 30 min. Subsequently, the DNA contents of each well of cells were stained with 100 μL Hoechst 33342 (5 μg/mL) for 30 min and visualized using a fluorescent microscope (Olympus, Japan). The red fluorescence indicated cells (EdU-positive) in the S phase of mitosis, and the blue fluorescence (Hoechst 33342 staining) indicated nuclei for identification of all cells. Cell proliferation was evaluated by determining the percentage of EdU-positive cells per 100 cells.

### Statistical analysis

All data were expressed as means ± standard deviations. Differences between groups were evaluated using analysis of variance (ANOVA), followed by Tukey’s multiple comparison test. SPSS15.0 statistical software was used for all analyses. Differences with *P* values of less than 0.05 were considered significant.

## Results

### Three-day exposure to hyperoxia induced CLD in newborn rats

**Fig. 1:**
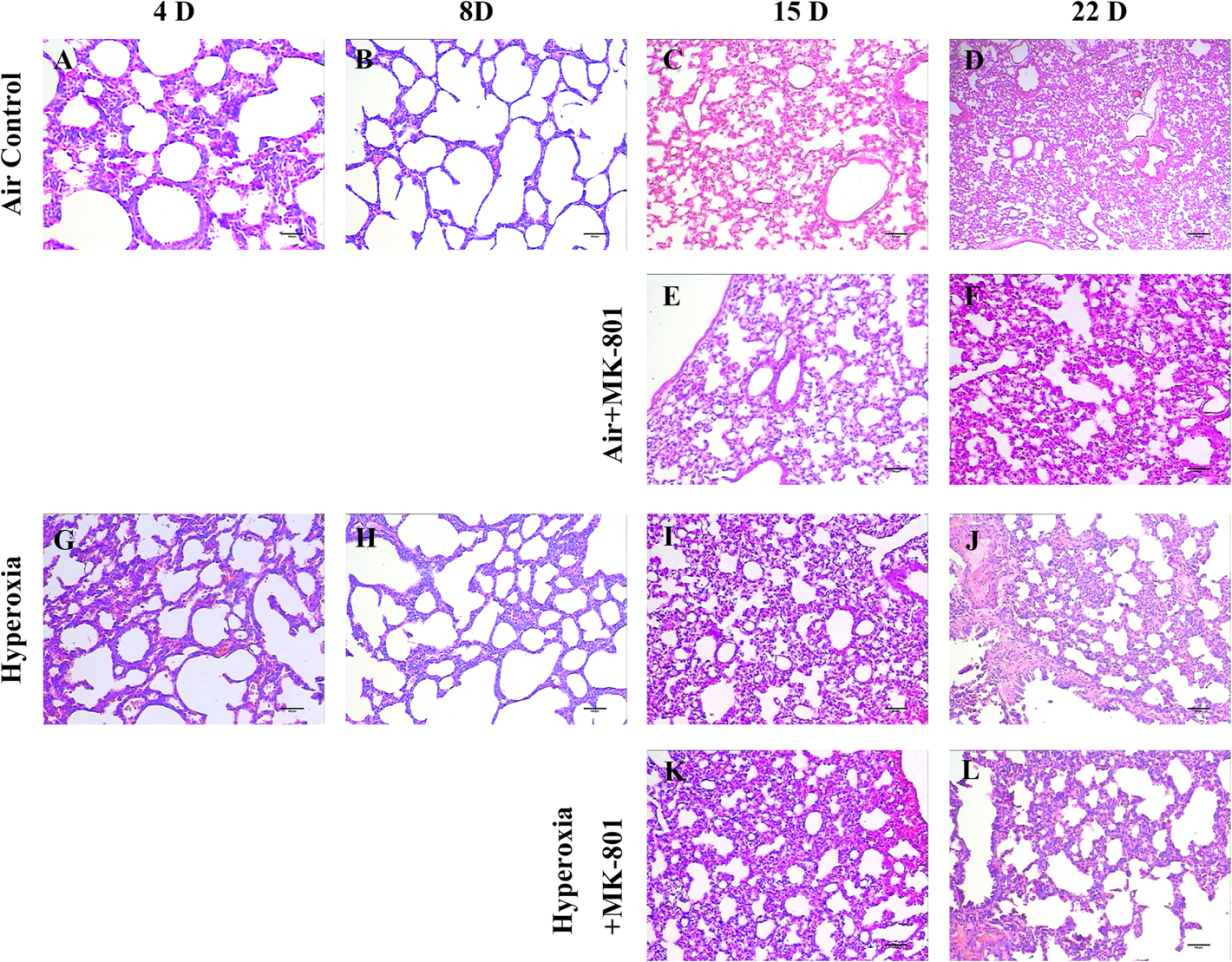
Lung histology. Normal development of alveolarization is seen in the control group (**a**-**d**). 3 days’ 95 % hyperoxia induced a small number of inflammatory cells in some of the alveolar spaces compared with the air control group at day 4 (**g** vs **a**). By day 8, inflammatory cells in the alveolar spaces had disappeared, and fewer alveoli were observed relative to the air group (**h** vs **b**). Wider alveolar septa and fewer alveoli were seen than in the air group on postnatal day 15 (**i** vs **c**). On postnatal day 22, alveolar septa had widened and alveolar numbers had decreased more markedly, and lung fibroblast proliferation could be observed in hyperoxia group (**j** vs **d**). MK-801 treatment in hyperoxia exposure rats showed thinner alveolar septa, greater numbers of alveoli, and a lower degree of lung fibroblast proliferations compared with the air group at postnatal days 15 and 22 (**k** and **l** vs **i** and **j**, respectively). MK-801 had no macroscopic effects on normal rats (**e** and **f** vs **c** and **d**, respectively). (Hematoxylin and Eosin × 40. Bar is 100 μm)

Lung histology revealed that there few inflammatory cells in some of the alveolar spaces in the hyperoxia group at day 4. By day 8, inflammatory cells in the alveolar spaces had almost disappeared, and fewer alveoli were observed relative to the air group on day 8. Additionally, wider alveolar septa and fewer alveoli were observed compared with those in the air group on postnatal day 15. On postnatal day 22, alveolar septa had widened, and alveolar numbers had decreased more markedly; additionally, lung fibroblast proliferation was observed (Fig. 1g–j versus a–d, respectively).

**Fig. 2:**
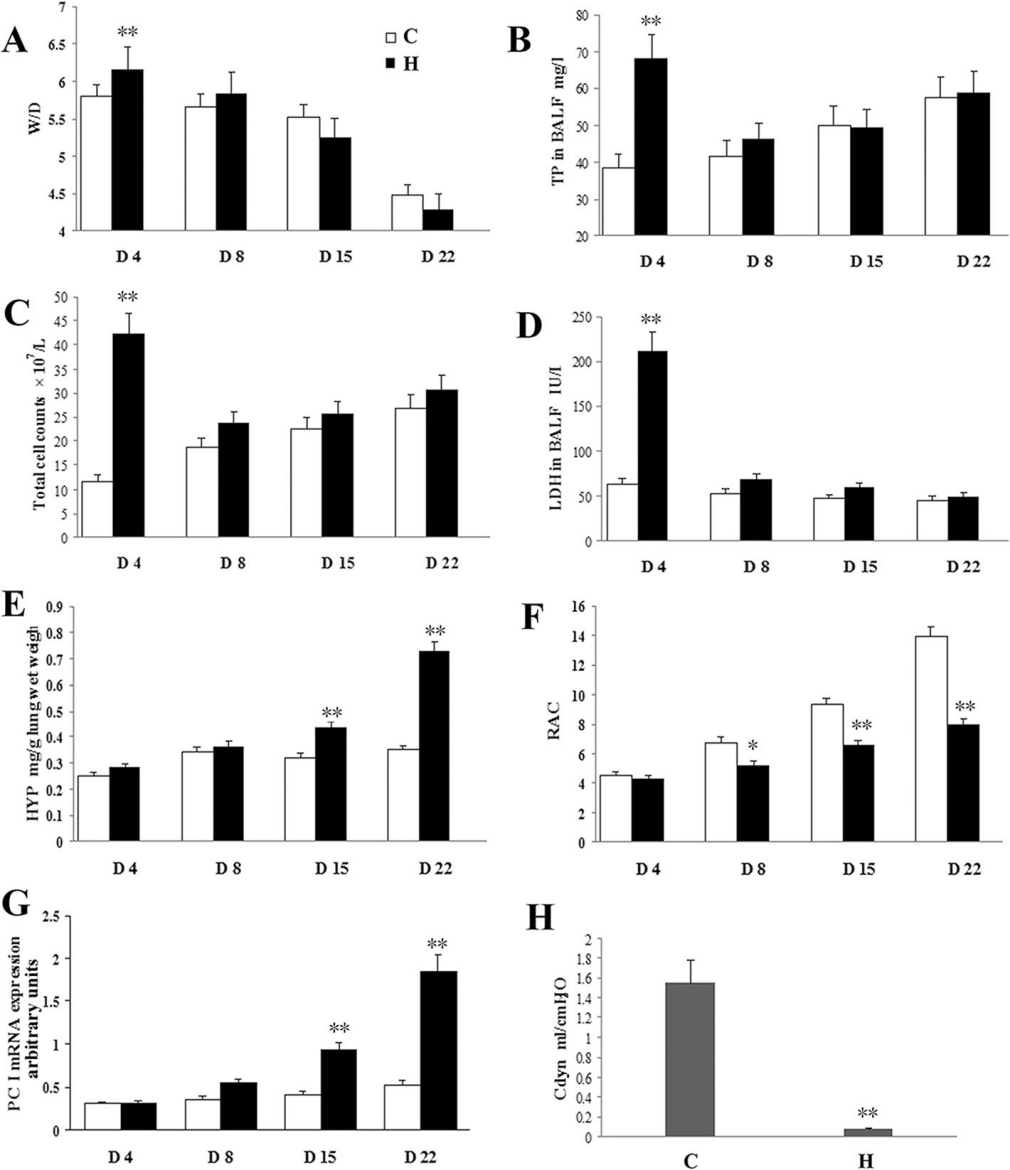
3 days’ exposure to hyperoxia induced CLD in newborn rats. At 4^th^ day, the W/D and the TP, LDH, and total cell counts in BALF of the hyperoxia group were significantly higher than in the air group (*P* < 0.01) (**a**-**d**). An obvious decrease 4 days after cessation of hyperoxia exposure (on postnatal day 8) was shown (*P* < 0.01). It was not different from the same day’s air control group (**a**-**d**). At day 8, there was a decline in RAC in the hyperoxia group compared with the air group (*P* < 0.05). RAC was also lower than in the air group at day 15 and 22 (*P* < 0.01) (**f**). At day 15 and 22, there was an increase in the level of HYP and the mRNA of type I procollagen in the hyperoxia group relative to the air group (*P* < 0.01) (**e** and **g**). A noticeable decrease in Cdyn in the hyperoxia group was recorded on day 22 (**h**). H: hyperoxia group; C: control group; CLD: chronic lung disease; BALF: bronchoalveolar lavage fluid; TP: Total protein; LDH: Lactate dehydrogenase activation; W/D: wet-weight/dry-weight ratios; HYP: hydroxyproline; Cdyn: dynamic lung compliance; RAC: Radial alveolar count; PC I: type I procollagen. *P <0.05, **P <0.01 vs. air group, *n* = 6 per group

After a 3-day exposure to hyperoxia, LDH, TP, total cell counts in BALF, and the W/D in the hyperoxia group were significantly higher than those in the air group (*P* < 0.01). These values showed obvious decreases 4 days after cessation of hyperoxia exposure (on postnatal day 8; *P* < 0.01) and were not different from those observed in the air control group on the same day (Fig. 2a–d).

On day 8, there was a decrease in RAC in the hyperoxia group compared with that of the air group (*P* < 0.05; Fig. 2f). The RAC was lower than that in the air group on postnatal days 15 and 22 (*P* < 0.01; Fig. 2f). On days 15 and 22, there was an increase in the level of HYP and the mRNA levels of type I procollagen in the hyperoxia group relative to those in the air group (*P* < 0.01; Fig. 2e and g). A noticeable decrease in Cdyn in the hyperoxia group was recorded on day 22 (Fig. 2h).

### MK-801 treatment during recovery from acute injury inhibited hyperoxia-induced pulmonary fibroplasia in newborn rats

Previous studies have shown that MK-801 treatment prevents hyperoxia-induced acute lung damage and airway inflammation in newborn SD rats [3, 11]. In order to rule out the anti-inflammatory effects of the NMDAR inhibitor on acute lung injury, MK-801 treatment was started 4 days after withdrawal of hyperoxia exposure (on postnatal day 8); at this stage, acute lung injury and inflammation had nearly disappeared, as described above (Fig. 2a–d).

Lung histology revealed that the hyperoxia + MK-801 group exhibited thinner alveolar septa, greater numbers of alveoli, and a lower degree of lung fibroblast proliferation than the air group on postnatal days 15 and 22 (Fig. 1k and l). MK-801 did not exert macroscopic effects in normal rats (Fig. 1e and f).

**Fig. 3:**
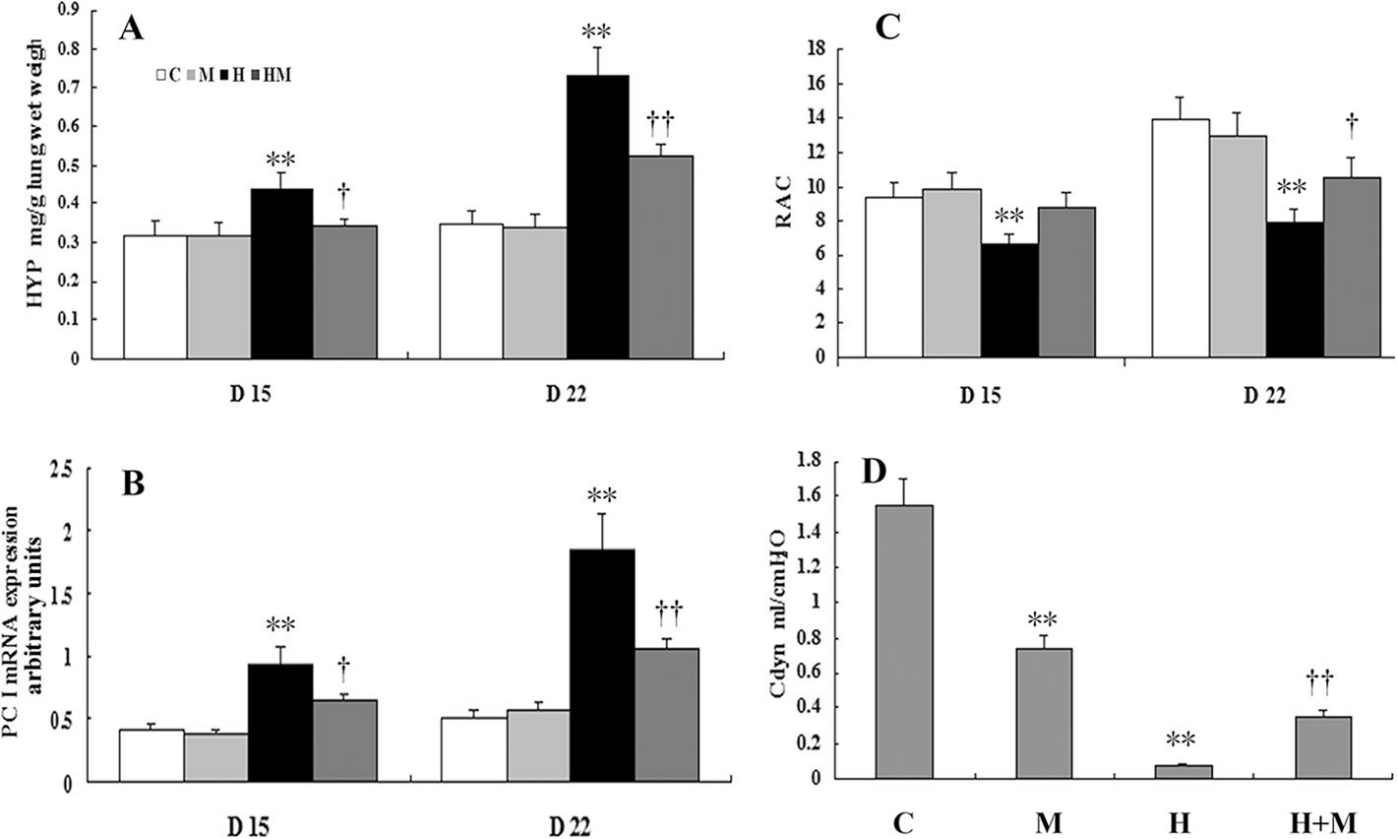
MK-801 treatment during recovery from acute injury inhibited hyperoxia-induced pulmonary fibroplasia in newborn rats. MK-801 treatment on days 8 to 10 in hyperoxia exposure rats exhibited less level of HYP (**a**) and type I procollagen mRNA expression (**b**), and a greater RAC (**c**) at days 15 and 22 (*P* < 0.05 or 0.01 respectively), as well as a greater improvement in Cdyn (*P* < 0.01) at day 22 (**d**) than the hyperoxia group. Moreover, MK-801 had no significant influence on HYP and RAC, but depressed Cdyn in normal rats (**d**). H: hyperoxia group; C: control group; M: air + MK-801 group; HM: hyperoxia + MK-801 group; HYP: hydroxyproline; Cdyn: dynamic lung compliance; RAC: Radial alveolar count; PC I: type I procollagen. **P <0.01 vs. air group; † *P* < 0.05, †† *P* < 0.01 vs. hyperoxia group, *n* = 6 per group

MK-801 treatment on postnatal days 8–10 in hyperoxia-exposed rats elicited lower levels of HYP and pro collagen I mRNA expression. Additionally, higher RACs were observed on days 15 and 22 (*P* < 0.05 or 0.01, respectively), and greater improvement in Cdyn (*P* < 0.01) was noted on day 22 compared with that in the hyperoxia group. In contrast, MK-801 had no significant influence on HYP and RAC, but lowered Cdyn in normal rats (Fig. 3a–d).

### Upregulation of NR2 mRNA in lung fibroblasts of hyperoxia-exposed newborn rats

The molecular and functional diversity of NMDARs is derived from at least seven gene family members that are alternatively spliced, leading to seven known types of subunits (NR1, NR2 [A–D], and NR3 [A–B]). The NR1 subunit allowed ions to permeate the heteromeric NMDA channel; the NR2 subunits, which are differentially expressed in various cell types, control the electrophysiological properties of the NMDAR [35, 36].

**Fig. 4:**
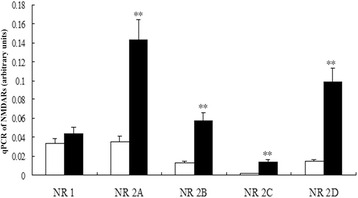
Upregulation of NR2 mRNA in lung fibroblasts of hyperoxia-exposed newborn rats. The mRNA levels of NR1 and the four NR2 subtypes (A, B, C, and D) in the lung fibroblasts of newborn rats were detected using real-time PCR. NR1 and NR2A were the dominant subtypes compared with other NR2 subtypes, NR2B and NR2D were moderately expressed, and NR2C expression was barely detectable. Hyperoxia had no influence on NR1, but upregulated the mRNA levels of all the NR2s (*P* < 0. 01), especially NR2A and NR2D. H: hyperoxia group; C: control group; NR1: NMDAR 1; NR2 A: NMDAR 2A; NR2 B: NMDAR 2B; NR2 C: NMDAR 2C; NR2 D: NMDAR 2D. **P <0.01 vs. air group, *n* = 6 per group

The mRNA levels of *NR1* and the four *NR2* subtypes (A, B, C, and D) in the lung fibroblasts of newborn rats were detected using real-time PCR. The results showed that *NR1* and *NR2A* were the dominant subtypes relative to other NR2 subtypes, *NR2B* and *NR2D* were moderately expressed, and *NR2C* expression was barely detectable. Hyperoxia had no influence on *NR1*, but upregulated the mRNA levels of all *NR2*s (*P* < 0. 01), particularly *NR2A* and *NR2D* (Fig. 4).

### NMDAR activation promoted the proliferation of lung fibroblasts and their differentiation into myofibroblasts in hyperoxia-exposed newborn rats

Myofibroblasts are metabolically and morphologically distinctive fibroblasts that express α-SMA; their activation plays a key role in the development of the fibrotic response. The expression of α-SMA correlates with the activation of myofibroblasts, which acquire new functions, such as a greater level of proliferation and collagen production than normal fibroblasts.

**Fig. 5:**
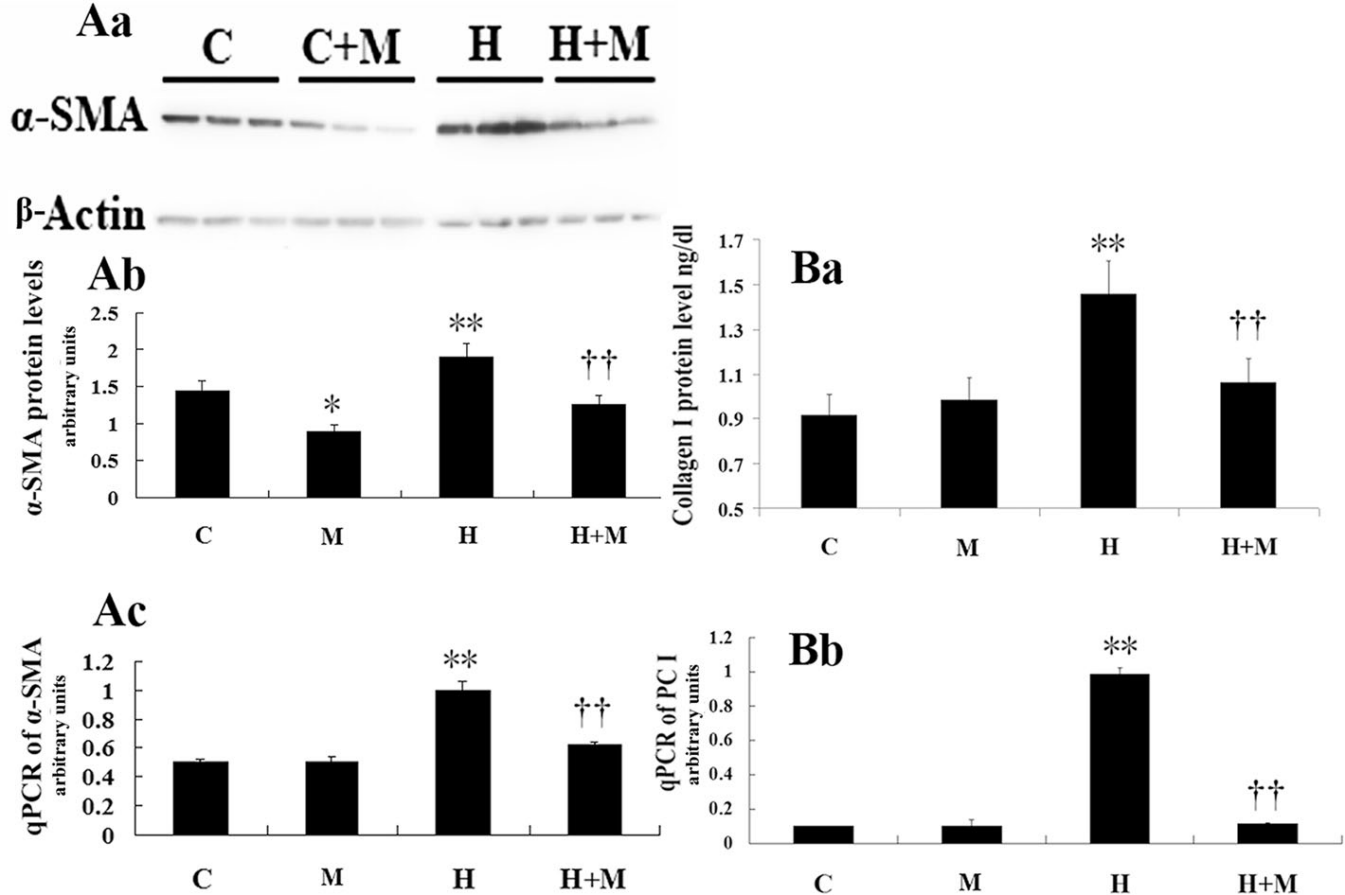
NMDAR activation upregulate lung fibroblasts α-SMA and collagen expression in hyperoxia exposure newborn rat. The protein levels (A a-b) and gene expression of α-SMA (A c) were significantly higher in cells stimulated with hyperoxia (*P* < 0.01) in newborn rat lung fibroblasts. The protein levels (B a) and gene expression of type I (pro) collagen (B b) were also higher in cells stimulated with hyperoxia (*P* < 0.01). Treatment with NMDAR antagonist MK-801 inhibited these changes (*P* < 0.01). PCI: type I procollagen. H: hyperoxia group; C: control group; M: air + MK-801 group; HM: hyperoxia + mk-801 group. **P <0.01 vs. air group, †† *P* < 0.05 vs. hyperoxia group, *n* = 6 per group

**Fig. 6:**
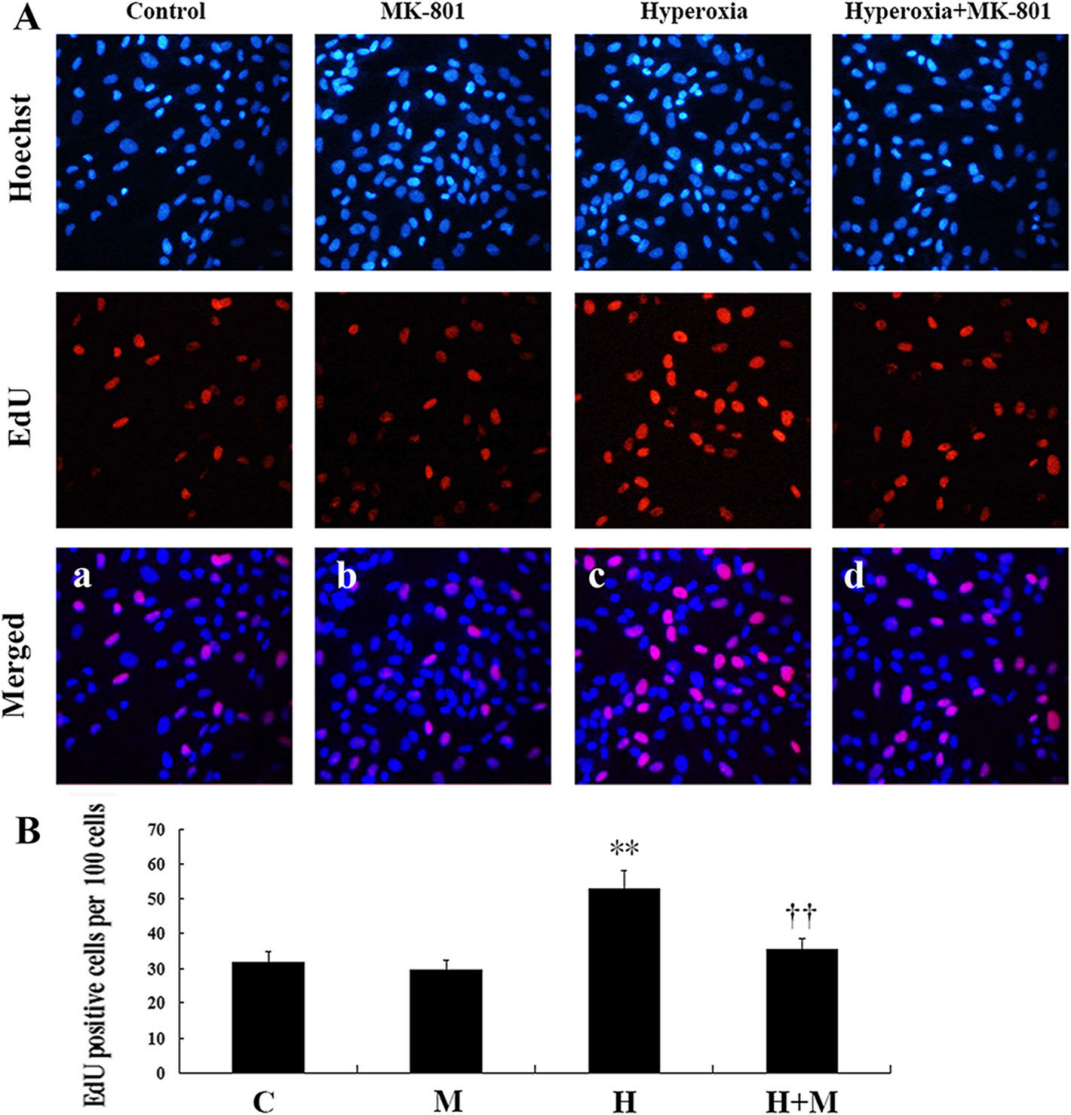
NMDAR activation promotes lung fibroblasts proliferation in hyperoxia exposure newborn rat. The red fluorescent cells (EdU-positive) were in the S phase of mitosis, and the blue fluorescent cells (Hoechst 33342-positive) represented all the cells. The percentage of EdU-positive cells was significantly higher in the hyperoxia group (*P* < 0.01) (A c vs A a, B), whereas MK-801 inhibited cells’ proliferation induced by hyperoxia (*P* < 0.01) (A d vs A c, B). MK-801 alone had no influence on normal cell growth. †† *P* < 0.05 vs. hyperoxia group, *n* = 6 per group; H: hyperoxia group; C: control group; M: air + MK-801 group; HM: hyperoxia + mk-801 group. Magnification × 20. **P <0.01 vs. air group

The gene expression and protein levels of α-SMA (Fig. 5A) and (pro) collagen I (Fig. 5B) were significantly higher in newborn rat lung fibroblasts stimulated by hyperoxia (*P* < 0.01). Treatment with the NMDAR antagonist MK-801 inhibited these changes (*P* < 0.01). The percentage of EdU-positive cells was significantly higher in the hyperoxia group (*P* < 0.01), whereas MK-801 inhibited the increase in the levels of EdU-positive cells induced by hyperoxia (*P* < 0.01). MK-801 alone had no influence on normal cell growth (Fig. 6).

## Discussion

Apparent pulmonary fibrosis, impaired alveolarization, and decreased pulmonary compliance were observed at postnatal days 15 and 22 after 3-day exposure to hyperoxia; however, acute lung inflammation and injury were nearly completely absent 4 days after cessation of hyperoxia. Our previous studies show that MK-801 treatment during acute lung injury improves lung histopathology and alveolarization and attenuates pulmonary ECM deposition in hyperoxia-induced lung injury in newborn rats via the inhibition of acute inflammation in the lung [3, 11]. In this study, MK-801 therapy initiated during the recovery period from acute injury improved alveolarization and pulmonary compliance and attenuated pulmonary ECM deposition manifested with the lower HYP and collagen expression. In this study, the expression of NMDARs was detected for the first time in vitro in lung fibroblasts of newborn rats. Hyperoxia upregulated the expression of four NR2s in the lung fibroblasts of newborn rats, stimulating cell proliferation and increasing the expression of α-SMA and type I (pro) collagen, although the cells were cultured during recovery from acute lung injury. MK-801 was found to inhibit these changes. These in vitro and in vivo findings indicated a possible mechanism underlying the morphologic transformation, via NMDAR activation, in hyperoxia-induced CLD in the lung fibroblasts of newborn rats.

Previous studies have shown that 1–3 days of 100 % hyperoxia exposure induces moderate lung inflammation and delayed alveolarization during recovery in newborn rats [15, 16]. The most important mechanism underlying hyperoxia-induced “old” BPD and pulmonary fibroplasia is the pulmonary acute inflammation induced by hyperoxia. However, over the last 15–20 years, studies have suggested that active cellular inflammation in the lung is not necessary for the development of pulmonary fibroses, such as idiopathic pulmonary fibrosis (IPF) [37]. In addition, severe acute lung injury and inflammatory reactions are not observed in most patients with BPD [1, 2]. These results coincide with our findings that 3-day hyperoxia exposure induced moderate inflammation and injury in the newborn rat lung and that these changes were nearly absent after stopping hyperoxia exposure for 4 days, progressing to aggravated apparent pulmonary fibrosis at postnatal days 15 and 22.

We have previously demonstrated that large amounts of endogenous glutamate are released in the lungs of newborn rats and that lung NR2D is strongly expressed after a 3-day exposure to hyperoxic conditions [3]. The NMDAR antagonist MK-801 decreases hyperoxia-induced acute lung damage [3, 11]. In this work, we showed that MK-801 therapy during recovery from acute lung injury (postnatal days 8–10) improves alveolarization and pulmonary compliance and attenuates pulmonary ECM deposition. The protective effects of MK-801 were attributable both to the inhibition of acute injury and inflammation and to chronic lung disease, as hyperoxia-induced acute lung injury and inflammation were nearly absent on postnatal day 8. These findings suggested that the activation of NMDAR during recovery from hyperoxia-induced lung injury played the same critical role as in acute lung injury.

Glutamate is the main ligand of NMDA receptors. However, the conflicting results of our previous studies showed that the content of Glu in the BALF decreases dramatically to a level below that of the air group after 7 days of hyperoxia exposure [2]. In contrast, the expression of NR2D in the hyperoxia group was much higher on day 7 than on days 1 or 3. Actually, persistent high extracellular glutamate levels are not observed in most brain injuries. For example, in patients with severe head injuries, intracerebral microdialysis often exhibits increased extracellular levels of glutamate in the brain only for a few hours to a few days after the primary insult [38, 39]. The initial increase in extracellular glutamate is cleared within 5 min after moderate traumatic brain injury, whereas antagonists of glutamate receptors remain effective when administered 30 min after insult [40]. These are consistent with our findings demonstrating that the content of Glu in the BALF decreased dramatically to a level below that of the air group at 7 days after hyperoxia exposure, while MK-801 therapy during recovery from acute lung injury improved hyperoxia-induced chronic lung injury. The mechanism of this incongruity between glutamate depletion and NMDAR activation in brain injury may involve glutamate receptor activation, which could help nervous tissue cope with reduced permeability of the cellular membrane to ions and increased efficacy of Na^+^ extrusion [40]. However, further studies are required to elucidate the underlying mechanism of NMDAR in hyperoxia-induced chronic lung injury.

The target cells of NMDAR inhibitors in hyperoxia-induced CLD are not known. The key processes of initiation and progression of lung fibrosis are thought to cause acute lung injury and inflammation, the epithelial-mesenchymal transition (EMT), fibroblast proliferation, and collagen deposition. Lung fibroblasts play a key role in lung development and repair following injury [17, 18]. Studies have shown that glutamate and NMDAR participate in cell differentiation and proliferation in the CNS [41–43], non-neuronal tissues, such as the retinal pigment epithelium (RPE) [44], and cancer cells [45]. In the last few years, NMDA-type glutamate receptors have been detected in fibroblasts. NMDARs modulate the expression of matrix metalloproteinase-2 in human fibroblast-like synoviocytes [25] and regulate cell differentiation in human periodontal ligament fibroblasts [27]. In the present study, NR1 and NR2 expression levels were detected in lung fibroblasts. Hyperoxia upregulated the four NR2s in lung fibroblasts in newborn rats, stimulating cell proliferation and increasing the expression of pro collagen I, whereas MK-801 inhibited these changes. These findings indicated that NMDAR activation in lung fibroblasts may play a role in hyperoxia-induced CLD in newborn rats.

Dysregulated myofibroblast development has been implicated in BPD [46, 47]. Although myofibroblasts are integral to normal repair mechanisms, the persistence of the myofibroblast beyond a period of normal repair has been shown to be associated with ECM deposition, structural remodeling, and destruction of alveocapillary units [48]. Lung myofibroblasts may be derived from peripheral blood fibrocytes and lung fibroblasts or from the pulmonary EMT; lung fibroblasts are a major source of lung myofibroblasts. However, the regulatory mechanisms of NMDAR activation involved in hyperoxia-induced pulmonary fibroblast transdifferentiation are poorly understood. In this work, we found that hyperoxia promoted the transdifferentiation of lung fibroblasts via the overactivation of NR2s, resulting in increased expression of α-SMA and improvement of cell proliferation and collagen deposition.

However, NR2 subunits, which are differentially expressed across various cell types, control the electrophysiological properties of the NMDAR [35, 36]. In this work, we observed upregulation of four NR2s in the lung fibroblasts of newborn rats. It is not known which NR2 receptor plays a key role in hyperoxia-induced transdifferentiation of lung fibroblasts. Additionally, lung myofibroblasts may also be derived from the pulmonary EMT. Indeed, NMDARs are expressed by alveolar epithelial cells [49]. However, we did not examine the role of NMDARs in the EMT in this study. Further studies of the various roles of the four NR2s in transdifferentiation of lung fibroblasts and the EMT are necessary for the development of therapeutic strategies against CLD and pulmonary fibrosis.

## Conclusion

In summary, the upregulation of NMDAR and the greater release of intrinsic glutamate in the neonatal rat lung after 3-day hyperoxia exposure [3], the protective role of MK-801 in hyperoxia-induced chronic lung injury, and the regulation of fibroblast transdifferentiation through overactivation of NR2s in lung fibroblasts indicated that NMDAR activation in lung fibroblasts plays important roles in hyperoxia-induced CLD in newborn rats. Our findings are expected to provide a therapeutic rationale for the treatment of infant CLD and other lung diseases that manifest as pulmonary fibrosis.

## Abbreviations

BALF: Bronchoalveolar lavage fluid
BPD: Bronchopulmonary dysplasia
Cdyn: Dynamic lung compliance
CLD: Chronic lung disease
ECM: Extracellular matrix
EdU: 5-ethynyl-29-deoxyuridine
HYP: Lung hydroxyproline
LDH: Lactate dehydrogenase activation
NMDARs: N-methyl-D-aspartate receptors
NR1: NMDAR 1
NR2A: NMDAR 2A
NR2B: NMDAR 2B
NR2C: NMDAR 2C
NR2D: NMDAR 2D
RAC: Radial alveolar count
TP: Total protein
W/D: Wet-weight/dry-weight ratio
α-SMA: Alpha-smooth muscle actin
